# Prefire grazing by cattle increases postfire resistance to exotic annual grass (*Bromus tectorum*) invasion and dominance for decades

**DOI:** 10.1002/ece3.2127

**Published:** 2016-04-12

**Authors:** Kirk W. Davies, Jon D. Bates, Chad S. Boyd, Tony J. Svejcar

**Affiliations:** ^1^ Eastern Oregon Agricultural Research CenterUSDA – Agricultural Research Service67826‐A Hwy 205BurnsOregon

**Keywords:** *Artemisia*, cheatgrass, disturbance, exotic annual grass, fire‐grazing interaction, herbivory, invasive plants, sagebrush

## Abstract

1. Fire, herbivory and their interaction influence plant community dynamics. However, little is known about the influence of prefire herbivory on postfire plant community response, particularly long‐term resistance to postfire exotic plant invasion in areas that historically experienced limited large herbivore pressure and infrequent, periodic fires.
2. We investigated the long‐term postfire effects of prefire herbivory by cattle, an exotic herbivore, in *Artemisia* (sagebrush) plant communities in the northern Great Basin, USA. Study areas were moderately grazed or not grazed by cattle since 1936 and then were burned in 1993. Plant community response was measured the 19th through the 22nd year postfire. Prior to burning exotic annual grass presence was minimal (<0.5% foliar cover) and plant community characteristics were similar between grazed and ungrazed treatments, with the exception of litter biomass being two times greater in the ungrazed treatment.
3. Two decades postfire, *Bromus tectorum* L., an exotic annual grass, dominated the ungrazed treatment. Native bunchgrasses, species richness, and soil biological crusts were greater in prefire grazed areas compared to ungrazed areas.
4. These results suggest that moderate prefire herbivory by cattle increased the resistance of the plant community to postfire invasion and dominance by *B. tectorum*. We presume that herbivory reduced mortality of large perennial bunchgrasses during the fire by reducing fine fuel (litter) and subsequently burn temperatures.
5. *Synthesis*: This research demonstrates that a moderate disturbance (herbivory) may mediate the effects of a subsequent disturbance (fire). The effects of disturbances are not independent; therefore quantifying these interactions is critical to preventing oversimplification of complex plant community dynamics and guiding the conservation of endangered ecosystems.

## Introduction

Fire and herbivory are well‐known drivers of plant community dynamics (Fuhlendorf and Engle 2004; Fuhlendorf et al. 2006; Kerby et al. 2007; Waldram et al. 2008). The effect of herbivory on fire effects is less well known and is increasingly of interest to understanding the herbivory‐fire interaction and its influence on plant communities (Davies et al. 2009; Kimuyu et al. 2014). Understanding grazing‐fire interactions is critical because many native plant communities are threatened by postfire exotic plant invasions. Furthermore, fire and grazing have independently been linked to invasion by exotic plants, especially exotic annual grass invasion in plant communities that are fire sensitive and did not recently evolve with high grazing pressure from large ungulates (Mack and Thompson 1982; Chambers et al. 2007, 2014).

Exotic annual grass invasions are degrading arid and semi‐arid ecosystems around the world (Purdie and Slatyer 1976; Mack 1981; Hobbs and Atkins 1988, 1990; D'Antonio and Vitousek 1992; Brooks et al. 2004; Milton 2004; Liu et al. 2006; Davies 2011). Invasion by exotic annual grasses is particularly concerning because they often increase fire frequency (D'Antonio and Vitousek 1992; Brooks et al. 2004; Balch et al. 2013) by increasing highly flammable fine fuel biomass and continuity (Davies and Nafus 2013). Increased fire frequency is an ecosystem level change that further facilitates invasion and perpetuates the dominance of exotic grasses (D'Antonio and Vitousek 1992; Rossiter et al. 2003). Exotic annual grass invasion also decreases biodiversity, degrades wildlife habitat, and reduces the economic productivity of rangelands (Davies and Svejcar 2008; Davies 2011).

One of the most problematic exotic annual grasses is *Bromus tectorum* L., which has invaded tens of millions of hectares in the *Artemisia* ecosystem of North America (Bradley and Mustard 2005; Meinke et al. 2009) and is found in all 50 US states and most Canadian provinces (NRCS 2015). Its invasion and dominance of large areas was facilitated by severe overgrazing by sheep, cattle, and horses following European‐American settlement (Mack 1981; Young and Allen 1997; Chambers et al. 2007) and more recently by fire (Knapp 1996; Chambers et al. 2007, 2014). Severe overgrazing reduces native perennial grasses (Young 1943; Laycock 1967; Young and Allen 1997), one of the most critical functional groups to resisting exotic annual grass invasion (Chambers et al. 2007; Davies 2008) because their temporal and spatial resource acquisition patterns overlap with exotic annual grasses (James et al. 2008). Thus, it is not surprising that *B. tectorum* dominance increases with sustained heavy livestock grazing in *Artemisia* communities (Reisner et al. 2013). Although, fire is a natural ecosystem driver in the *Artemisia* ecosystem that shifts dominance from woody vegetation to herbaceous vegetation (Wright and Bailey 1982; Mensing et al. 2006), the introduction of exotic annual grasses has resulted in a threat of converting these communities to annual grasslands postfire. *Bromus tectorum* often increases after fire; probably because of increased resource availability with the loss of fire intolerant shrubs and fire‐induced mortality of perennial bunchgrasses (Stubbs and Pyke 2005; Chambers et al. 2007; Davies et al. 2007, 2014a).

Millions of acres of *Artemisia* rangelands, however, are grazed by domestic livestock and/or burn in wildfires without transitioning to exotic annual grasslands. Rangelands with well‐managed livestock grazing in the *Artemisia* ecosystem generally have similar vegetation characteristics as ungrazed areas including limited exotic annual grass abundance (West et al. 1984; Rickard 1985; Courtois et al. 2004; Manier and Hobbs 2006). Davies et al. (2007) and Rhodes et al. (2010) found that burning *Artemisia tridentata* ssp. *wyomingensis* with a relatively intact perennial bunchgrass understory did not result in significant invasion of *B. tectorum*. In contrast, Stewart and Hull (1949) reported exotic annual grass invasion and dominance increased following burning when the perennial bunchgrass understory had been degraded prefire. This suggests that postfire exotic annual grass invasion is highly correlated with prefire plant community composition (i.e., the abundance of perennial bunchgrasses). However, if fire‐induced mortality of perennial bunchgrasses is high, then relatively intact prefire communities may have significant *B. tectorum* invasion postfire (Davies et al. 2009). The combination of low perennial bunchgrass abundance postfire and the release of nutrients with fire appear to greatly elevate the risk of exotic annual grass invasion and dominance. Supporting this hypothesis, Chambers et al. (2007) found that removing herbaceous perennial vegetation or burning increased cheatgrass biomass and seed production between two and six times, but the combination of both removal and burning resulted in increases between 10 and 30 times.

Clearly, overgrazing facilitates *B. tectorum* invasion and dominance by adversely affecting mechanisms mediating invasion resistance (Mack 1981; Young and Allen 1997; Reisner et al. 2013). Indubitably, fire can increase the risk of *B. tectorum* invasion and dominance (D'Antonio and Vitousek 1992; Knapp 1996; Chambers et al. 2007). Grazing, however, may mediate fire severity by altering fuel characteristics (Davies et al. 2010, 2015; Kimuyu et al. 2014). Davies et al. (2016) found that moderate prefire grazing reduced maximum temperature and duration of elevated temperatures (heat loading) at the meristematic crown of perennial bunchgrasses during a fire. Similarly, in Kenya, grazing by wildlife and by cattle prefire lowered burn temperatures compared to ungrazed areas (Kimuyu et al. 2014). Reduced temperatures and less heat loading during a fire decrease the likelihood of fire‐induced mortality of perennial bunchgrasses (Wright and Klemmedson 1965; Wright 1970; Odion and Davis 2000; Pelaez et al. 2001). However, successive disturbances often have compounding effects (Paine et al. 1998) and disturbance interactions can have a synergistic impact on exotic plant invasion (Hobbs and Huenneke 1992). The interaction between livestock grazing and fire, therefore, may be more critical to postfire resistance to *B. tectorum* invasion and dominance than either factor individually. *Bromus tectorum* invasion can be transient or represent a threshold in plant community composition (Bagchi et al. 2013). Therefore, long‐term evaluation is needed to better understand the influence of moderate prefire grazing on postfire resistance to *B. tectorum* invasion and dominance as well as native plant community dynamics.

The purpose of this study was to determine long‐term (two decades) postfire plant community dynamics in shrub‐grasslands that were either moderately grazed by an exotic herbivore, cattle, or not grazed for +50 years prior to burning. We accomplished this task by resampling the study plots of Davies et al. (2009) 19 through 22 years postfire. We postulated that long‐term moderate grazing prefire increases the resistance of the native plant community to postfire exotic annual grass invasion and dominance. Thus, we expect that (1) *B. tectorum* cover, density, and biomass would be less, (2) large perennial bunchgrass cover, density, and biomass would be greater, (3) plant species richness and diversity would be greater, and (4) biological soil crust cover would be greater in areas moderately grazed prefire compared to ungrazed areas two decades after burning.

## Materials and Methods

### Study area

The study was conducted in southeastern Oregon at the Northern Great Basin Experimental Range (NGBER) (lat 43°29′N, long 119°43′W) about 56 km west of Burns, Oregon, USA. Climate at the NGBER is typical of the northwestern Great Basin with hot, dry summers and cool, wet winters. Average annual precipitation at the NGBER was 264 mm over the past 30 years (1980–2010) (PRISM 2015). Annual precipitation was 92%, 69%, 61%, and 105% of the long‐term average in 2011, 2012, 2013, and 2014, respectively (PRISM 2015). Soils at the study sites are loamy mixed, frigid, shallow Aridic Durixerolls, coarse‐loamy, mixed frigid, Orthidic Durixerolls, and coarse‐loamy Aridic Duric Haploxerolls. Two of the replicates were Droughty Loam 11‐13 PZ (R023XY316OR) and one was Clayey 10‐12 PZ (R023XY220OR) Ecological Site. Elevation across the study area is approximately 1400 m above sea level and topography is generally flat (slopes 0–3°). *Artemisia tridentata* ssp. *wyomingensis* was the dominant woody species at all sites prior to burning and dominant grass species varied among sites. *Achnatherum thurberianum* (Piper) Barkworth, *Pseudoroegneria spicata* (Pursh) A. Löve, *Festuca idahoensis* Elmer, *Koeleria macrantha* (Ledeb.) J.A. Schultes, and *Elymus elymoides* (Raf.) Swezey were common large perennial bunchgrasses across study sites. *Poa secunda* J. Presl, a smaller and earlier developing perennial bunchgrass, was also common at the study sites. Common perennial forbs included *Phlox longifolia* Nutt., *Crepis* L. sp., *Astragalus* L. sp., *Erigeron* L. sp., *Eriogonum* Michx. sp., *Lomatium* Raf. sp., and *Achillea millefolium* L. These plant communities recently evolved with few large herbivores (Mack and Thompson 1982) and cattle, an exotic herbivore, were introduced in large numbers to this region in the mid to late 1800s (Oliphant 1968). Native ungulates were not abundant and rarely used the study area. *Artemisia tridentata* ssp. *wyomingensis* communities of the Intermountain West are estimated to have fire return intervals of >100 years (Mensing et al. 2006). The study sites had no evidence of burning for 50 years prior to exclosure construction and did not burning during the experiment other than the applied controlled burns.

### Experimental design

A randomized block design was used to evaluate the effects of prefire grazing on long‐term postfire plant community dynamics. Treatments were applied at three different sites (blocks) with varying soils and vegetation characteristics. Treatments were: (1) grazed and (2) ungrazed by cattle for +50 years prior to being burned. In 1936, three 2‐ha exclosures were constructed to apply the ungrazed treatment in three different 65 ha pastures. Exclosures were four‐strand barb‐wire fences and native herbivores were not excluded. Grazed treatment plots were established adjacent to each exclosures in the same plant communities on the same soil types. In 1937, densities of large perennial bunchgrasses, *P. secunda*, perennial forbs, annual grasses, and annual forbs did not differ between treatments (Davies et al. 2009). Livestock grazing pressure in the grazed treatment was moderate, 30–40% consumption of available forage, and was applied until 1990 (Davies et al. 2009). From 1938 to 1949 cattle use was rotation grazing with stocking rates determined from range production surveys conducted in 1938 and 1944. Then from 1949 until 1990, the grazing system was a deferred‐rotational system with infrequent years of complete rest. Grazing timing of use altered between April‐June (growing season use) and July‐August (deferred use). Grazing was excluded 2 years prior to burning to accumulate enough fuel to carry the fire across the study plots. Cover, density, and annual biomass of herbaceous species did not differ between treatments prior to burning in 1992 and 1993 (Rose et al. 1994). Herbaceous litter biomass was almost two times greater in the ungrazed compared to the grazed treatment prior to burning (Rose et al. 1994). Herbaceous fuel load prior to burning averaged across all sites was 689 kg·ha^−1^ and 793 kg·ha^−1^ in grazed and ungrazed treatments, respectively (Rose et al. 1994). Prescribed burns were applied as strip‐head fires using drip‐torches to 0.4 ha plots in each treatment at each site in late September of 1993. Weather conditions during the prescribed burns were variable: relative humidity varied between 8 and 22%, air temperature ranged from 20 to 27°C, and wind speeds were between 6 and 21 km·h^−1^. Burns were complete across study sites with 100% mortality of sagebrush.

### Measurements

Vegetation characteristics were sampled in June of 2012–2015, the 19th through the 22nd year post‐fire. Each treatment at each site was sampled with a 30 × 60‐m plot centered in the treatment area to limit edge effects. Herbaceous cover and density were measured in 0.2‐m^2^ quadrats located at 3‐m intervals along five 30‐m transects resulting in 10 quadrats per transect and 50 quadrats per plot. Ground cover was visually estimated based on markings that divided the quadrats into 1, 5, 10, 25, and 50% segments. Litter, bare ground, and soil biological crust cover were also estimated in the 0.2‐m^2^ quadrats. The 30‐m transects were spaced parallel to each other at 15‐m intervals. Shrub cover by species was measured using the line‐intercept method (Canfield 1941) on the five 30‐m transects. Shrub density by species was measured by counting all shrubs rooted in five 2 × 30‐m belt transects located along the five 30‐m transects in each plot. Plant species richness and diversity (Shannon Diversity Index) were determined from density measurements (Krebs 1998). Annual herbaceous biomass was determined by functional group by harvesting 25 randomly located 1‐m^2^ quadrats in each plot. Harvested biomass was oven dried to a constant weight, current and previous years' growth separated, and current year's growth was then weighed.

### Statistical analysis

Repeated‐measures analysis of variance (ANOVA) using the mixed models procedure (Proc Mixed SAS v. 9.1; SAS Institute, Cary, NC) was used to determine the effects of moderate levels of grazing prefire on long‐term plant community dynamics postfire. Year was the repeated variable, treatment was considered a fixed variable, and site and site by treatment interaction were treated as random effect variables in analyses. Akaike's Information Criteria were used to select the appropriate covariance structure for repeated‐measures ANOVAs (Littell et al. 1996). Herbaceous vegetation was separated into five groups for analyses: *P. secunda*, perennial bunchgrasses, perennial forbs, *B. tectorum*, and annual forbs. *Poa secunda* was treated as a separate functional group because it is smaller, develops earlier (James et al. 2008), and responds to disturbances differently than other native perennial bunchgrasses in the *Artemisia* ecosystem (McLean and Tisdale 1972; Winward 1980; Yensen et al. 1992). *Bromus tectorum* was the only exotic annual grass species detected at the study plots. Data that did not meet assumptions of ANOVAs were either log or square‐root transformed. Original data (i.e., nontransformed) are presented in figures and text. Means are reported with standard errors in figures and text. Means were considered different at *P *≤* *0.05.

## Results

**Figure 1:**
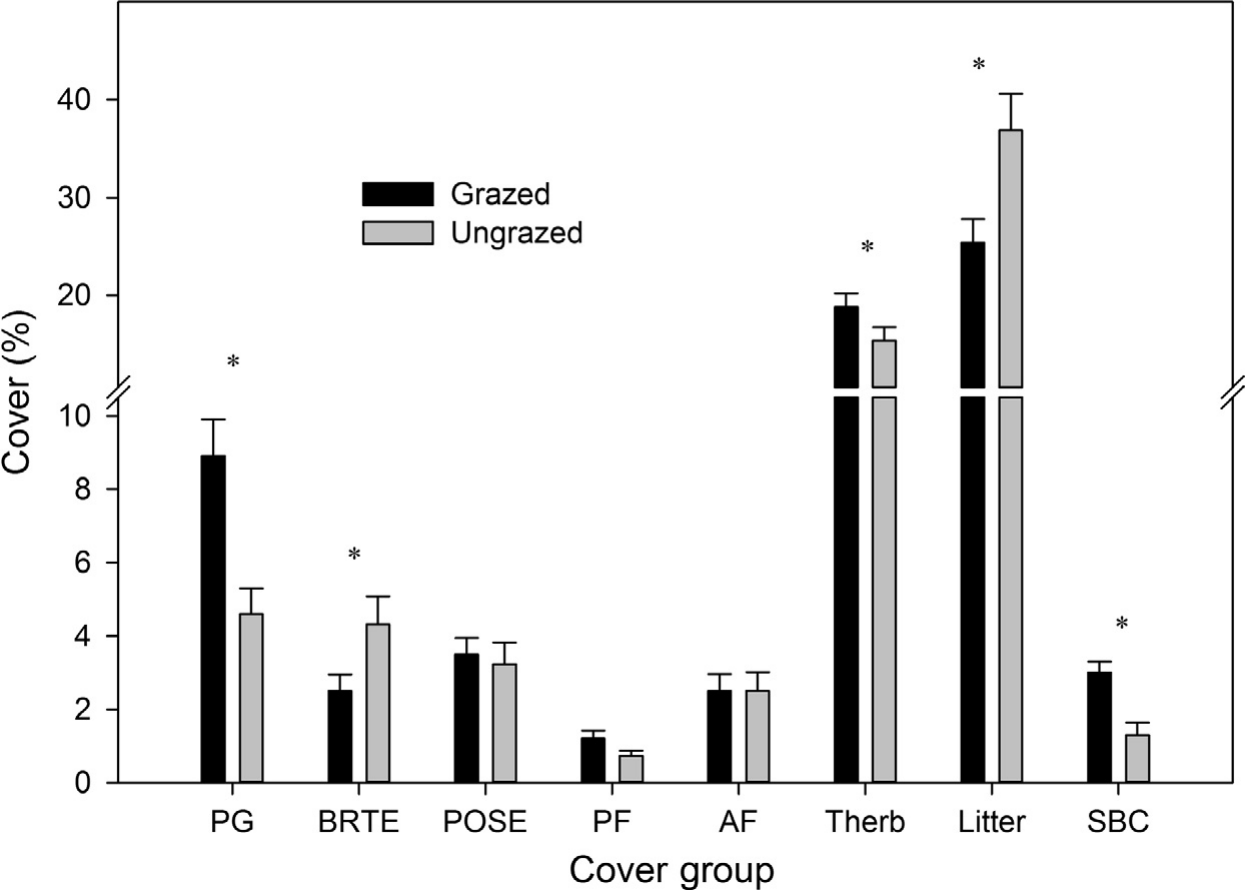
Cover (mean + SE) of cover groups 19–22 years postfire in areas that were grazed or ungrazed by cattle for +50 years pre‐fire. PG = large perennial bunchgrasses, BRTE = *Bromus tectorum*, POSE = *Poa secunda*, PF = perennial forbs, AF = annual forbs, Therb = total herbaceous, Litter = ground litter, and SBC = Soil biological crusts. Asterisk (*) denotes significant (*P < *0.05) difference between treatments.

Large perennial bunchgrass and *B. tectorum* cover varied by treatment (Fig. 1; *P *=* *0.022 and 0.026). Averaged across all years, large perennial bunchgrass cover was 1.9‐fold greater in the grazed compared to the ungrazed treatment. *Bromus tectorum* cover was 1.7‐fold greater in the ungrazed compared to the grazed treatment. *Poa secunda*, perennial forb, and annual forb cover did not differ between treatments (Fig. 1; *P *=* *0.782, 0.210, and 0.844). Total herbaceous cover was 1.2 times greater in the grazed compared to the ungrazed treatment (Fig. 1; *P *=* *0.012). Litter, predominately comprised of previous years' *B. tectorum* growth, was 1.5‐fold greater in the ungrazed compared to the grazed treatment (Fig. 1; *P *=* *0.012), but bare ground did not differ between treatments (data not presented; *P *=* *0.150). Soil biological crust ground cover was 2.3 times greater in the grazed compared to the ungrazed treatment (Fig. 1; *P *=* *0.019). Sagebrush cover did not differ between treatments (data not presented; *P *=* *0.261). We did not find evidence that the interaction between treatment and year was significant for any of the measured cover response variables (*P *>* *0.05).

**Figure 2:**
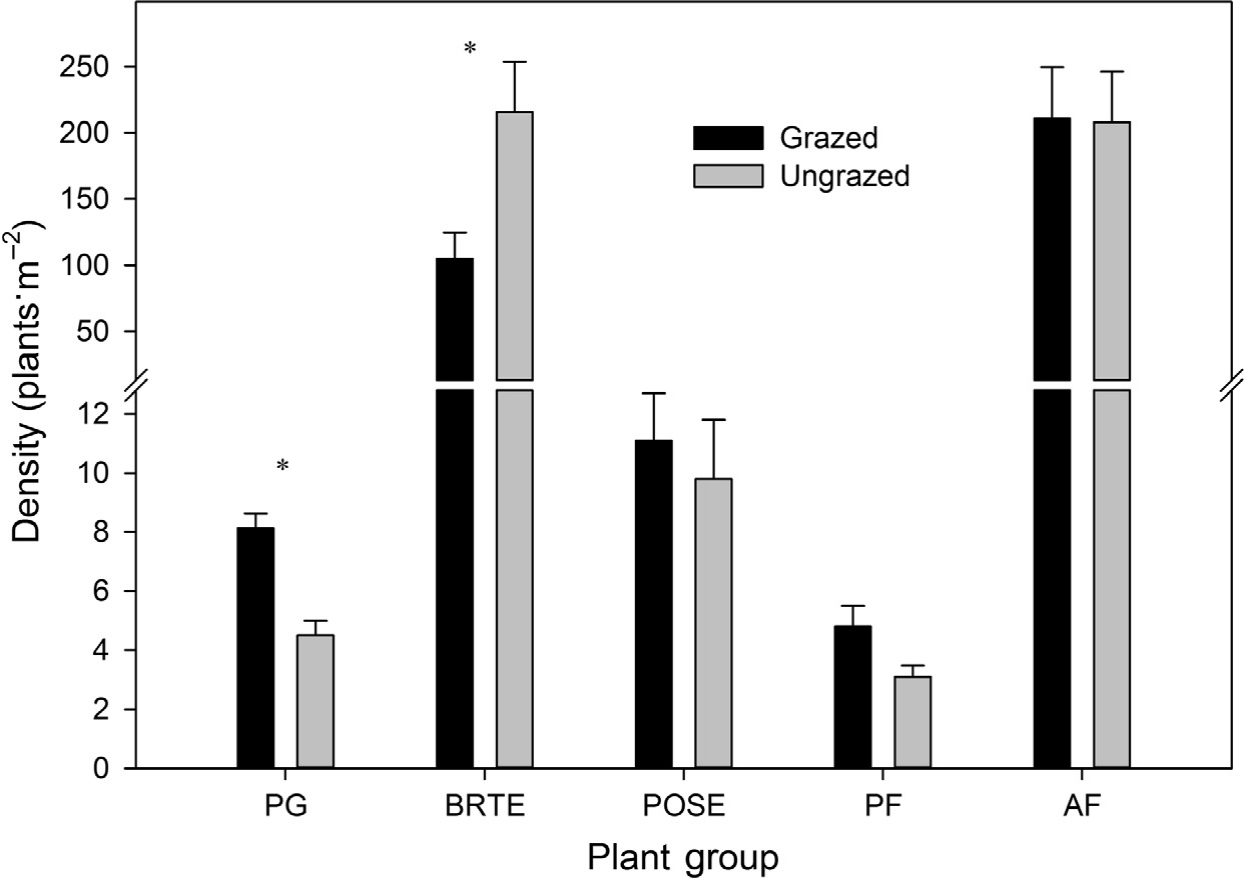
Density (mean + SE) of plant groups 19–22 years postfire in areas that were grazed or ungrazed by cattle for +50 years prefire. PG = large perennial bunchgrasses, BRTE = *Bromus tectorum*, POSE = *Poa secunda*, PF = perennial forbs, and AF = annual forbs. Asterisk (*) denotes significant (*P < *0.05) difference between treatments.

Areas that were grazed prefire had large perennial bunchgrass densities that were 1.8‐fold greater than ungrazed areas (Fig. 2; *P *=* *0.009). Grazing prefire decreased *B. tectorum* density postfire (Fig. 2; *P *=* *0.023). *Bromus tectorum* density was 2.1 times greater in the ungrazed compared to the grazed treatment. The density of *P. secunda*, perennial forbs, and annual forbs did not differ between grazed and ungrazed treatments (Fig. 2; *P *=* *0.716, 0.305, and 0.930). We did not find evidence that sagebrush density varied between treatments (data not presented; *P *=* *0.197). The interaction between treatment and year was not significant for any of the measured density response variables (*P *>* *0.05).

**Figure 3:**
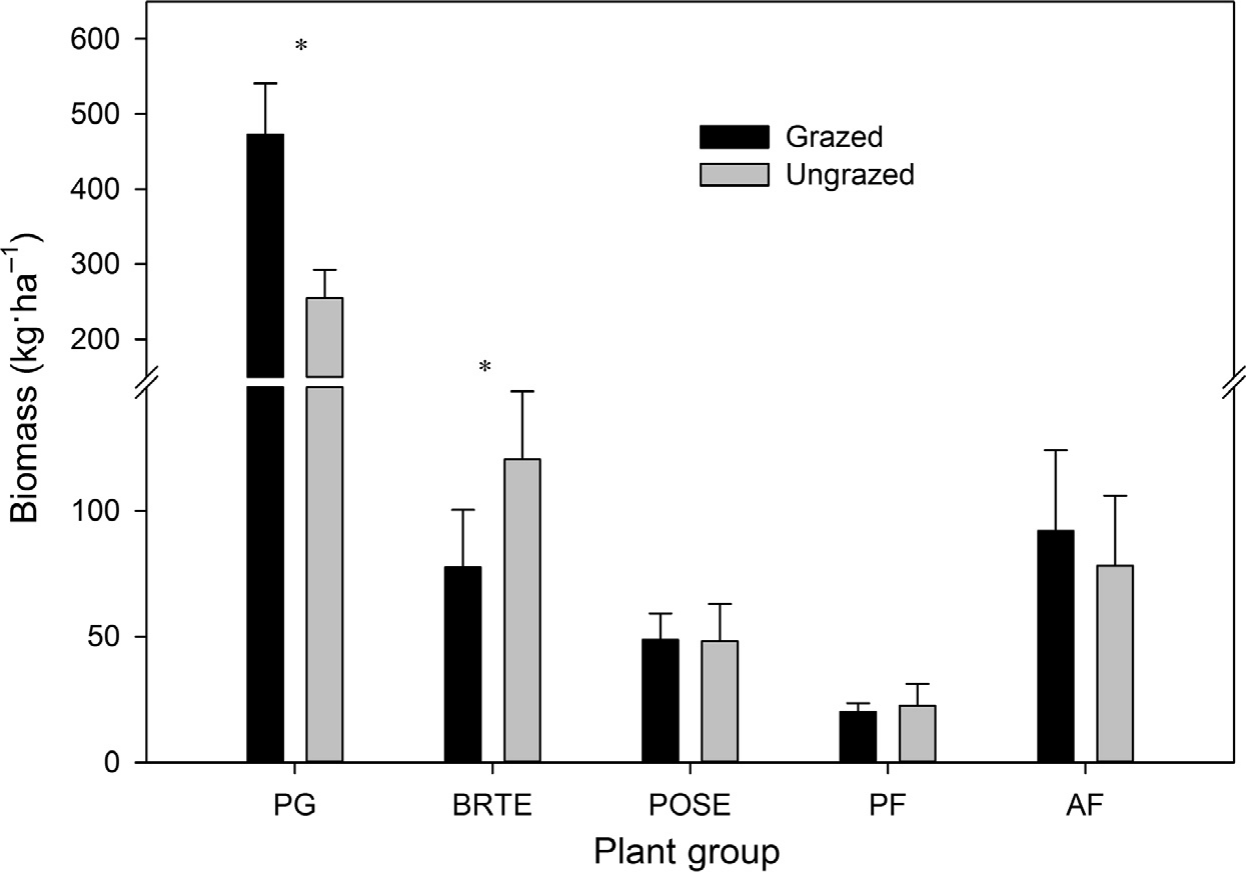
Biomass (mean + SE) of plant groups 19–22 years postfire in areas that were grazed or ungrazed by cattle for +50 years prefire. PG = large perennial bunchgrasses, BRTE = *Bromus tectorum*, POSE = *Poa secunda*, PF = perennial forbs, and AF = annual forbs. Asterisk (*) denotes significant (*P < *0.05) difference between treatments.

Biomass of large perennial bunchgrasses was 1.9‐fold greater in the grazed compared to the ungrazed treatment (Fig. 3; *P *=* *0.005). *Bromus tectorum* biomass was 1.6 times greater in the ungrazed than the grazed treatment (Fig. 3; *P *=* *0.002). *Poa secunda*, perennial forb, and annual forb biomass did not vary between treatments (Fig. 3; *P *=* *0.980, 0.828, and 0.774). The interaction between year and treatment was not significant for biomass for any measured response variables (*P *>* *0.05).

Total species richness varied between treatments (*P *=* *0.033). The grazed treatment (20.6 ± 1.1 species·plot) had on average two more species per plot than the ungrazed treatment (18.7 ± 0.75 species·plot). Shannon's diversity index did not differ between the grazed (1.23 ± 0.09) and ungrazed (1.17 ± 0.06) treatment (*P = *0.636). The interaction between treatment and year did not influence total species richness or Shannon's diversity index (*P *=* *0.309 and 0.502).

## Discussion

Moderate grazing by an exotic herbivore (cattle) increased native plant community resistance to post‐fire *B. tectorum*, an exotic annual grass, invasion. The postfire invasion and dominance by *B*.* tectorum* in the ungrazed area are evident +20 years postfire suggesting that these are not transient responses and are a fundamental shift in the plant community that is unlikely to be easily reversed. The results of this study suggest that the effects of grazing and fire, and presumably other disturbances, are not independent and thus quantifying interactions is critical. It has been well established that effects of grazing by cattle vary considerably by intensity, frequency, and timing (Bates and Davies 2014; Davies et al. 2014b). However, many studies still report grazing as a binomial treatment; either it is grazed or not grazed and grazing intensity, frequency and timing are not reported (Jones 2000; Borman 2005). The results of the present study suggest that effects of grazing by cattle in the *Artemisia* ecosystem of North America and likely rangelands globally are probably oversimplified even when grazing intensity, frequency, and timing are considered. The influence of grazing on fire effects is just as important, or more important, than the direct effects of grazing on vegetation.

Moderate grazing by cattle likely altered the response of *Artemisia* plant communities to fire via its effects on fuel characteristics and subsequently fire severity. In support of this postulation, Kimuyu et al. (2014) reported that cattle grazing can reduce fuel loads and thereby lower fire temperatures in savannas. Prior to burning, fuel loads were greater in ungrazed compared to grazed areas (Rose et al. 1994) and in a nearby study, fine fuel loads were twice as high in long‐term grazing exclosures compared to adjacent grazed areas (Davies et al. 2010). Probably most critical, fuel biomass can be three times greater on perennial bunchgrasses in exclosures compared to adjacent moderately grazed areas (Davies et al. 2010). Greater fine fuels likely increased fire temperatures and the duration of elevated temperatures during the burn (Byram 1959; Davies et al. 2016). Greater temperatures and long periods of elevated temperatures during a fire increase the risk of mortality of perennial herbaceous vegetation (Wright and Klemmedson 1965; Wright 1970; Odion and Davis 2000; Pelaez et al. 2001). Fewer perennial bunchgrasses in ungrazed areas after fire further suggests that fire‐induced mortality was greater in the ungrazed treatment compared to the moderately grazed treatment. Hence, the lack of herbivory in the ungrazed treatment resulted in an accumulation of fine fuels on perennial bunchgrasses that likely led to their lower abundance postfire.

Prefire grazing positively altered the resistance of these plant communities because abundance and cover of perennial bunchgrasses and soil biological crusts were twofold greater in grazed compared to ungrazed areas postfire. The loss of perennial bunchgrasses in the ungrazed treatment when it burned decreased the resistance of the plant community to *B. tectorum* invasion and dominance because perennial bunchgrasses are one of the most important plant functional groups limiting exotic annual grass invasion (Chambers et al. 2007; Davies 2008) as their resource acquisition patterns overlap with exotic annual grasses (James et al. 2008). Decreases in soil biological crusts in ungrazed areas are also concerning because soil biological crusts are an important ecosystem component that capture soil resources, reduce erosion, and increase resistance to invasion in arid and semi‐arid plant communities (Belnap et al. 2001; Harper and Belnap 2001; Belnap 2006). Reductions in soil biological crust are also concerning because their decline is correlated with increases in exotic annual grasses (Ponzetti et al. 2007; Dettweiler‐Robinson et al. 2013). Our results are counter to the general assumption that livestock negatively impact soil biological crusts and also provide additional insight into the relationship between livestock grazing and soil biological crusts. Prior research has demonstrated that soil biological crusts decrease with increased livestock activity (Ponzetti and McCune 2001; Yeo 2005) and fire (Hilty et al. 2004; Ponzetti et al. 2007). However, our research suggests that livestock grazing influence on fire may positively affect soil biological crusts. Moderate grazing, by reducing fire severity (Davies et al. 2016), is probably mediating fire effects on soil biological crust.

Twice the density, cover, and biomass of *B. tectorum* in the ungrazed treatment compared to the grazed treatment two decades postfire demonstrates that postfire resistance to exotic annual grass invasion and dominance was lower in ungrazed areas. Ungrazed areas shifted from perennial dominance to codominance between exotic annuals and perennial herbaceous vegetation after burning. The conversion to annual grass dominated rangeland is likely a permanent transition to a new steady state as it is costly and difficult to restore *A. tridentata* ssp. *wyomingensis* plant communities (Davies et al. 2011). Currently, there are no cost‐effective techniques to even control *B. tectorum* over the large acreages it has invaded (Stohlgren and Schnase 2006). The invasion and dominance of rangelands by exotic annual grasses also increase the likelihood of frequent fires (Balch et al. 2013; Davies and Nafus 2013) and subsequent, further degradation as an annual grass‐fire cycle develops (D'Antonio and Vitousek 1992; Brooks et al. 2004).

The magnitude of difference in *B. tectorum* between grazed and ungrazed treatments was considerably less than reported a decade after fire (Davies et al. 2009). *Bromus tectorum* has significantly increased in the grazed treatment in the last decade, but remained approximately the same in the ungrazed treatment. We speculate that this is the result of high *B. tectorum* propagule pressure from the ungrazed area undermining the resistance of the prefire grazed treatment. Ecological resistance to exotic invasion can be overwhelmed by propagule pressure (Holle and Simberloff 2005; Fensham et al. 2013). The ungrazed treatments were adjacent to the grazed treatments at each site and therefore, the high abundance of *B. tectorum* in the ungrazed area over the last decade likely resulted in high propagule pressure in the grazed treatment. The last several years have also been unusually favorable for *B. tectorum* in the northern Great Basin, with it having high abundance on sites where it prior had limited abundance (EOARC file data). The abundance of large perennial bunchgrasses, the most important plant group to resistance to exotic annual grass invasion (Chambers et al. 2007; Davies 2008), has maintained the same twofold difference in the grazed compared to the ungrazed treatment reported in Davies et al. (2009). However, perennial grass cover was ~20% less in the grazed compared to a decade ago, likely due to increased competition from *B. tectorum*. Though we cannot quantify the exact impact of the grazed treatment being immediately adjacent to the heavily exotic annual grass‐invaded ungrazed treatment, it unquestionably has negatively impacted the native plant community and increased the abundance of *B. tectorum*.

The results of our study appear to be in contrast with the Reisner et al. (2013) finding that grazing by cattle exacerbates *B. tectorum* dominance in the *Artemisia* steppe ecosystem. The differing conclusions drawn from our research and Reisner et al. (2013) probably relate to two factors. Reisner et al. (2013) did not account for fire effects in their analysis and, consequently, could not evaluate the interactive effects of grazing and fire. Secondly, we only evaluated moderate grazing which has been repeatedly demonstrated to have nominal effects on *Artemisia* plant communities in the absence of fire (West et al. 1984; Rickard 1985; Courtois et al. 2004; Manier and Hobbs 2006). Negative effects from grazing observed by Reisner et al. (2013) were likely due to sustained heavy use (i.e., overgrazing). This infers that statements such as grazing exacerbates exotic annual grass dominance in the *Artemisia* ecosystem and that cumulatively reducing cattle grazing will restore this ecosystem (Reisner et al. 2013) are not fully supported when a more holistic view of ecosystem dynamics is considered. There is no question that repeated heavy grazing decreases the resistance of the *Artemisia* ecosystem to exotic annual grass invasion. However, our data suggest that moderate grazing, through its influence on fire effects, can have the opposite effect.

The postfire response of the ungrazed areas implies that suggestions to remove or greatly reduce exotic herbivores, predominantly domestic cattle, to conserve the *Artemisia* ecosystem (e.g., Beschta et al. 2013; Reisner et al. 2013) may, at times in certain locations, further degrade this ecosystem. Careful management of grazing by livestock, nevertheless, is required to prevent undesirable shifts in vegetation (Daubenmire 1940; Mack and Thompson 1982; Reisner et al. 2013) since defoliation reduces photosynthetic tissue and can place defoliated plants at a competitive disadvantage with nondefoliated plants (Caldwell et al. 1987; Briske and Richards 1995; Holechek et al. 1998). Conversely, grazing exclusion allows fuels to accumulate, which may result in greater fire risk (Davies et al. 2010, 2015) and fire‐induced mortality of biological soil crusts and bunchgrasses critical to plant community resistance to exotic annual grass invasion and dominance.

Though effects of allowing herbaceous fuels to accumulate may not be realized immediately or even in the next couple of decades, fires are a natural disturbance in the *Artemisia* ecosystem that shifts dominance from woody vegetation to herbaceous vegetation (Wright and Bailey 1982; Mensing et al. 2006) and inevitably these plant communities will burn (Davies et al. 2012). However, it would be naive to assume that all ungrazed *Artemisia* plant communities will convert to exotic annual grasslands postfire. The probability of exotic annual grass invasion and dominance postfire is likely elevated in some of these plant communities, but whether or not this occurs will also depend on a multitude of other factors, such as fire weather, fuel characteristics not influenced by grazing, plant community composition, pre and postfire weather, postfire management, site environmental characteristics, and other variables influencing resistance to exotic annual grass invasion (Chambers et al. 2007, 2014; Davies et al. 2011).

Managing the resistance of rangeland plant communities to postfire exotic plant invasion will almost certainly become more critical in the future. Almost all models predict increased wildfire frequency with climate change (Westerling et al. 2006; Fulé 2008; Yue et al. 2013). These wildfires are also predicted to become more severe (Fried et al. 2004), which probably heightens the risk of postfire exotic plant invasion, especially by exotic annual grasses. Management will need to account for the interaction between prefire grazing and fire on postfire plant community dynamics. Fine fuel management may be critical in rangeland ecosystems to manage fire risk and severity (Davies et al. 2009, 2010; Strand et al. 2014) and subsequently, native plant communities resistance to exotic plant invasion.

There is a wide range of vegetation, soil and environmental characteristics across the *Artemisia* ecosystem (Davies et al. 2006; Davies and Bates 2010; Chambers et al. 2014). Similarly, wildfires burn across a broad range of conditions and postfire weather can be variable. These factors likely interact among themselves and with grazing to determine postfire vegetation response. The response of *Artemisia* plant communities to disturbance can vary with climatic and environmental characteristics (Davies et al. 2011; Nelson et al. 2014). Similarly, other rangeland ecosystems are highly variable and plant community dynamics are influenced by a broad array of interacting factors. Research investigating the interaction between grazing and fire characteristics across the range of other potential explanatory variables would be invaluable to better understand the influence of grazing in this ecosystem and other ecosystems and to develop appropriate management strategies to sustain native rangelands.

This research demonstrates that interactions between disturbances can be critical to plant community resistance to exotic plant invasion and dominance. Too often, the effects of a disturbance are oversimplified because its interaction with other disturbances is overlooked. Other research has also observed that prior disturbance influences subsequent disturbance effects (e.g., Veblen et al. 1994; Kulakowski and Veblen 2002, 2007; Platt et al. 2002). These studies found that prior disturbances generally magnify the effects of subsequent disturbance. Paine et al. (1998) determined that successive disturbances, multiple disturbances that occur in short succession, compound their effects. In contrast, our results suggest that prior disturbances may also mediate subsequent disturbance effects. Moderate grazing by cattle, which alters fuel structure, increased the resistance of the native plant community to post‐fire exotic annual grass invasion. Our study and others (Veblen et al. 1994; Paine et al. 1998; Kulakowski and Veblen 2002, 2007; Platt et al. 2002) demonstrate that disturbance history has a substantial influence on effects of succeeding disturbances.

## Conclusions

Our research demonstrates the need to quantify the effects of interactions among disturbances. Prior disturbances or lack of it can alter the subsequent disturbance impact on plant communities; possibly result in transitions to new states that will be difficult to reverse. In our study, prefire herbivory reduced postfire exotic annual grass invasion for over two decades, while grazing exclusion resulted in a transition to an exotic annual grass‐invaded state. Moderate grazing by cattle, an exotic herbivore, increased native plant community resistance to exotic annual grass invasion and dominance, likely by altering fuel structure and consequently heat loading. Disturbances outside the historical disturbance regime (e.g., grazing by an exotic herbivore) are often assumed to negatively impact native plant communities. However, this research suggests disturbances outside the historical disturbance regime may in some circumstances positively influence native plant communities, in this situation through its impact on another disturbance. Obviously, the effects of grazing, fire and other disturbances and their interactions will vary by a suit of factors. The effects of disturbances are not independent and identifying and quantifying these interactions is critical to better understand complex plant community dynamics and conserve endangered ecosystems.

## Conflict of Interest

None declared.
